# Evaluation of Antioxidant and Free Radical Scavenging Capacities of Polyphenolics from Pods of *Caesalpinia pulcherrima*

**DOI:** 10.3390/ijms13056073

**Published:** 2012-05-18

**Authors:** Feng-Lin Hsu, Wei-Jan Huang, Tzu-Hua Wu, Mei-Hsien Lee, Lih-Chi Chen, Hsiao-Jen Lu, Wen-Chi Hou, Mei-Hsiang Lin

**Affiliations:** 1 Graduate Institute of Pharmacognosy, School of Pharmacy, Taipei Medical University, 250 Wuxing St., Taipei 11031, Taiwan; E-Mails: hsu0320@tmu.edu.tw (F.-L.H.); wjhuang@tmu.edu.tw (W.-J.H.); lmh@tmu.edu.tw (M.-H.L.); m303090001@tmu.edu.tw (H.-J.L.); wchou@tmu.edu.tw (W.-C.H.); 2 Department of Pharmacy, Taipei Medical University Hospital, 250 Wuxing St., Taipei 11031, Taiwan; 3 School of Pharmacy, College of Pharmacy, Taipei Medical University, 250 Wuxing St., Taipei 11031, Taiwan; E-Mails: thwu@tmu.edu.tw (T.-H.W.); lcchen@health.gov.tw (L.-C.C.); 4 Department of Pharmacy, Taipei City Hospital, No.145, Zhengzhou Rd., Taipei 10341, Taiwan

**Keywords:** 1,1-diphenyl-2-picrylhydrazyl (DPPH) radical, hydroxyl radical, peroxynitrite, dihydrorhodamine 123 (DHR 123), butylated hydroxytoluene (BHT)

## Abstract

Thirteen polyphenolics were isolated from fresh pods of *Caesalpinia pulcherrima* using various methods of column chromatography. The structures of these polyphenolics were elucidated as gallic acid (**1**), methyl gallate (**2**), 6-*O*-galloyl-d-glucoside (**3**), methyl 6-*O*-galloyl-β-d-glucoside (**4**), methyl 3,6-di-*O*-galloyl-α-d-glucopyranoside (**5**), gentisic acid 5-*O*-α-d-(6′-*O*-galloyl)glucopyranoside (**6**), guaiacylglycerol 4-*O*-β-d-(6′-*O*-galloyl)glucopyranoside (**7**), 3-methoxy-4-hydroxyphenol 1-*O*-β-d-(6′-*O-*galloyl) glucopyranoside (**8**), (+)-gallocatechin (**9**), (+)-catechin (**10**), (+)-gallocatechin 3-*O*-gallate (**11**), myricetin 3-rhamnoside (**12**), and ampelopsin (**13**). All isolated compounds were tested for their antioxidant activities in the 1,1-diphenyl-2-picrylhydrazyl (DPPH), hydroxyl, and peroxynitrite radicals scavenging assays. Among those compounds, **11**, **12**, and **2** exhibited the best DPPH-, hydroxyl-, and peroxynitrite radical-scavenging activities, respectively. Compound **7** is a new compound, and possesses better scavenging activities towards DPPH but has equivalent hydroxyl radical scavenging activity when compared to BHT. The paper is the first report on free radical scavenging properties of components of the fresh pods of *Caesalpinia pulcherrima*. The results obtained from the current study indicate that the free radical scavenging property of fresh pods of *Caesalpinia pulcherrima* may be one of the mechanisms by which this herbal medicine is effective in several free radical mediated diseases.

## 1. Introduction

Reactive oxygen species (ROS), major free radicals generated in many redox processes [1], often induce oxidative damage to biological molecules, such as carbohydrates, lipids, proteins, and DNA. Many serious diseases and accelerated aging, including cardiovascular diseases, inflammation, and neurodegenerative diseases are caused by biomolecular degeneration, followed by the initiation and propagation of oxidative chain reactions [2,3]. Thus, it has been suggested that antioxidant compounds may prevent aging by scavenging free radicals and delaying or preventing oxidation of biological molecules [4]. Plants containing phenolic ingredients, such as phenolic acids, phenolic diterpenes, flavonoids, tannins, and coumarins, are potential sources of natural antioxidants [5]. Numerous studies have revealed that these natural antioxidants possess multiple pharmacological activities, including neuroprotective, anticancer, and anti-inflammatory activities, and that these activities may be related to their antioxidant properties [6]. The importance of flavonoids in foods means that it is indispensable to have suitable methods of determining their content. Several studies have focused on separation methodology in order to quantify flavonoids [7–9] and simultaneously determine flavonols and anthocyanins [10]. The impacts of specific conditions including light exposure, field curing, freeze-drying process on the flavonols content of plant foods [10–16] have also been documented.

*Caesalpinia pulcherrima* Swartz (Leguminosae) is a commonly used medicinal herb in Taiwan. Different parts of this herb are used in common remedies to treat a number of disorders including menoxenia, pyrexia, bronchitis, wheezing, and malarial infection. A recent study of this folk remedy showed that it possesses antimicrobial [17], anti-tubercular [18], antiviral [19], antiulcer [20], anti-inflammatory [20,21], cytotoxic [17,22], and antioxidant [17,22,23] activities. Isolated compounds from published studies on the *C. pulcherrima.* include diterpenoids [24–29], homoisoflavonoids [30–32], flavonoids [30,31], chalcones [31], and peltogynoids [30].

The MeOH/CH_2_Cl_2_ (1:1) crude extracts from flower and leaf parts of *C. pulcherrima* have been evaluated by the DPPH (1,1-diphenyl-2-picrylhydrazyl)-scavenging method [23]. However, the individual bioactive compounds of *C. pulcherrima* were not isolated. In this study, we were interested in the antioxidant activity of pods of *C. pulcherrima*. Herein, we report on the isolation, structural elucidation, and antioxidative results of isolated compounds (**1**~**13**) from the acetone extract of fresh pods of *C. pulcherrima*. The antioxidant potential of the isolated compounds was evaluated by DPPH-, hydroxyl-, and peroxynitrite-scavenging assay methods.

## 2. Results and Discussion

### 2.1. Isolation of Phenolic Compounds of *C. pulcherrima*

**Figure 1:**
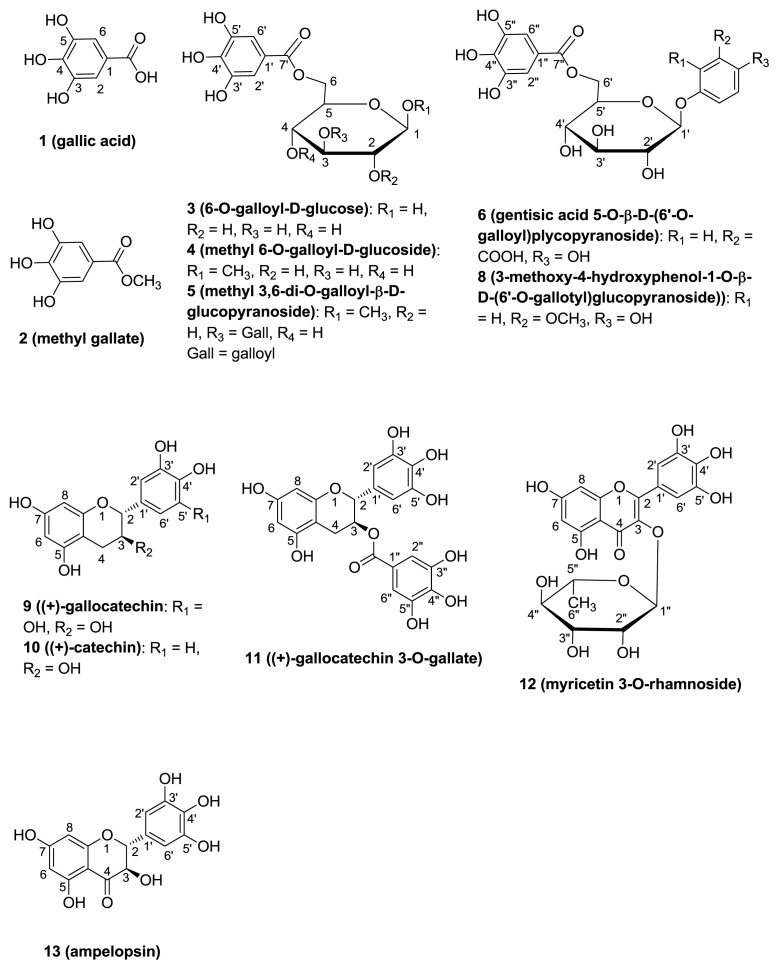
Structures of isolated compounds **1**~**6** and **8**~**13**.

**Figure 2:**
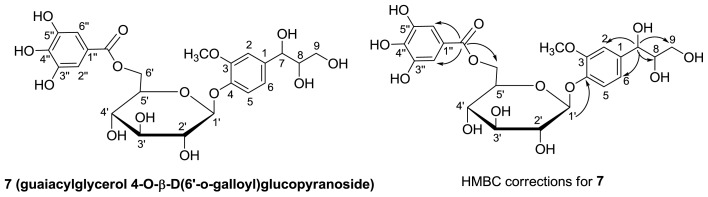
Structure and HMBC corrections of compound **7**.

Thirteen polyphenols shown in Figures 1,2 were obtained from an 80% aqueous acetone extract of *C. pulcherrima* pods using various methods of column chromatography. By comparison to the previous literature, compounds **1**~**6** and **8**~**13** were identified as known compounds including two simple polyphenols, gallic acid (**1**) [33] and methyl gallate (**2**) [34]; three gallotannins of 6-*O*-galloyl- d-glucoside (**3**) [35,36], methyl 6-*O*-galloyl-α-d-glucoside (**4**) [37], and methyl 3,6-di-*O*-galloyl-α-d-glucopyranoside (**5**) [36]; two phenol glycoside gallates of gentisic acid 5-*O*-α-d-(6′-*O*-galloyl) glucopyranoside (**6**) [38] and 3-methoxy-4-hydroxyphenol 1-*O*-β-d-(6′-*O*-galloyl) glucopyranoside (**8**) [38,39]; three flavan-3-ols of (+)-gallocatechin (**9**) [40], (+)-catechin (**10**) [41], and (+)-gallocatechin 3-*O*-gallate (**11**) [41]; one flavonol of myricetin 3-rhamnoside (**12**) [42]; and one dihydroflavonol of ampelopsin (**13**) [43]. Flavonoids **9** and **12** are major compounds of *C. pulcherrima* pods and yield 0.007% (0.62 g).

Compound **7** was obtained as colorless needle-like crystals, and its molecular formula was determined to be C_23_H_28_O_14_ based on HR-FAB-MS data at *m*/*z* 551.1381 [M+Na]^+^ (calcd. for C_23_H_28_NaO_14_, 551.1377). Its infrared (IR) spectrum indicated the presence of a hydroxyl group (3368 cm^−1^), a conjugated carbonyl functional group (1698 cm^−1^), and aromatic rings (1616 cm^−1^). The ultraviolet (UV) spectrum also showed the presence of aromatic rings (277 nm). The optical rotation was [α]_D_^20^ −14.1° (c 1.0, MeOH). ^1^H NMR indicated the presence of a galloyl group (*δ* = 7.13, 2H, s), ABX type aromatic signals (*δ* = 7.10, 1H, d, 8.4 Hz; *δ* = 7.05, 1H, d, 1.8 Hz; *δ* = 6.85, 1H, dd, 8.4 and 1.8 Hz), and a methoxy group (*δ* = 3.80, 3H, s), together with a sugar moiety (*δ* = 3.40~4.93). Compound **7** was assigned as galloyl-d-glucoside skeleton by comparison with compound **3** in the pattern of ^1^H NMR and ^13^C NMR spectra. The ^13^C NMR and HMQC spectra interpreted that each proton was correlated with a carbon (Table 1). The H-H COSY spectrum showed correlations of H-5↔H-6, H-1′↔H-2′, H-1′↔H-5′↔H-6′, and H-7↔H-8↔H-9. The HMBC showed correlations of δ 4.57 (H-7) with *δ* 63.9 (C-9), 76.9 (C-8), 112.0 (C-2), and 120.2 (C-6), which suggested that the glycerol moiety was connected to the C-1 position (Figure 2). In addition, correlations of *δ* 166.7 (C-7″) with *δ* 7.13 (H-2″, H-6″), 4.63, and 4.32 (H-6′) and *δ* 4.92 (H-1′) with *δ* 146.8 (C-4) indicated that gallate C-7″ and phenyl glycerol C-4 were respectively attached to sugars at C-6′ and C-1′. Based on the data described above, the structure of compound **7** was elucidated as guaiacylglycerol 4-*O*-β-d-(6′-*O*-galloyl)glucopyranoside.

### 2.2. Antioxidant Activities

#### 2.2.1. DPPH Radical-Scavenging Effect

The DPPH assay is a simple and quick test, and commonly used to assess the antioxidant activities of natural plants and compounds which act as free radical scavengers *in vitro*. Thus, we evaluated the free radical-scavenging activity of 13 polyphenols from *C. pulcherrima* pods using the above assay. All test compounds exhibited appreciable scavenging properties against the DPPH radical, and the inhibition percentage was proportional to the concentration of each compound. All compounds showed better antioxidative activities than the positive control (BHT), and the highest DPPH-scavenging activity was shown by compound **11**, followed by compounds **5**, **2**, and **13** (Table 2). The IC_50_ value for compound **11** was 3.3 μM, which was 5-fold more potent than BHT (16.57 μM). Compounds **1**~**3** and **9**~**12** had been investigated on DPPH-scavenging activities previously [44–47]. The IC_50_ value for gallic acid (**1**) was 7.59 μM in the DPPH assay, and this data is similar to that reported in Yokozawa’s study (IC_50_ value 8.14 μM) [44]. The IC_50_ value for methyl gallate (**2**) was 4.62 μM in the DPPH assay, and this data is similar to that reported in Pfundstein’s study (IC_50_ value 4.28 μM) [45]. DPPH-scavenging activities of other compounds are similar to prior results, in the present studies.

From these results, it was also possible to make a number of correlations regarding the relationship between the structure of isolated compounds and their DPPH-scavenging activities. Methyl ester (**2**) appeared to enhance the bioactivity of gallic acid (**1**). Glycosylation of gallic acid enhanced the DPPH-scavenging activities as with gallotannins **3**~**5**, and bioactivity increased in accordance with the increased number of galloyl groups. It was found that the antioxidant activity of gallotannins and simple phenols decreased in the following sequence: **5** > **2** > **4** > **3 > 1**. It appears that as far as the antioxidant activity is concerned, a galloyl group is essential, while ester and glycoside gallates have greater bioactivity. Comparing the DPPH-scavenging activities of the glycoside gallates (**3** and **4**) with phenol glycoside gallates **7**~**9** revealed that glycoside gallates with 1-phenoxy and 1-methoxy had higher levels of activity, and the bioactivity decreased in the following sequence: **4** > **6**~**8** > **3**. This suggests that the π-donor of the phenoxy and inductive electron-donating methoxy substituent made a greater IC_50_ (concentration of each substance (μM) when 50% of a radical is scavenged) contribution to the antioxidant activity than did the hydroxyl at position 1 of the glycoside gallate. The antioxidant activity of phenol glycoside gallates substituted in the benzene ring of 1-glycosyl group was affected through substitution of methoxy, carboxyl, hydroxyl, or 2′,3′-dihydroxypropyl, and the bioactivity decreased in the following sequence: **7** > **8** > **6** (*i.e.*, 2-methoxy-4(2′,3′-dihydroxypropyl) > 3-methoxy-4-hydroxyl > 3-carboxy-4-hydroxyl). It was found that the antioxidant activity of flavan-3-ols decreased in the following sequence: **11** > **10** > **9** (*i.e.*, 3-*O*-gallate, 5′-OH > 3-OH, 5′-H > 3-OH, 5′-OH). This suggests that the presence of the gallate group at the 3 position plays the most important role in the free radical-scavenging abilities. An additional insertion of the hydroxyl group at the 5′ position in the B ring also contributes to the scavenging activities. Comparing the DPPH-scavenging activity of flavan-3-ols (**9** and **10**), flavonol **12**, and dihydroflavonol **13** revealed that the 4-carbonyl group had a higher level of activity, and the bioactivity decreased in the following sequence: **13** > **12** > **10** > **9**. It was found that the antioxidant activity of the isolated compounds decreased in the following sequence: **11** > **5** > **2** > **13** > **12** > **4** > **7** > **8** > **10** > **6** > **3** > **1** > **9**. This confirmed that more phenol groups lead to an increase in antioxidant activity [44].

#### 2.2.2. Hydroxyl Radical-scavenging Activity

ROS constitute a major pathological factor causing many serious diseases, including cancer and neurodegenerative disorders [2]. The generally formed ROS are oxygen radicals, such as hydroxyl radicals and superoxide, and non-free radicals, such as hydrogen peroxide and singlet oxygen [48]. The hydroxyl radical is the most reactive and induces severe damage to adjacent biological molecules. Thus, the hydroxyl radical-scavenging activity of isolated compounds was investigated using the 2-deoxyribose method. All compounds exhibited appreciable scavenging properties against hydroxyl radicals, and the inhibition percentage was proportional to the concentration of each compound. No tested compound showed better antioxidative activity than the positive control (BHT), and the highest hydroxyl-scavenging activity was observed for compound **12**, followed by compound **7** (Table 2). The respective IC_50_ values for compound **12** and **7** were 0.78 and 0.89 μM and they had similar potencies to BHT. To our knowledge, the abilities of the compounds **1**, **2**, and **13** to scavenge hydroxyl radicals were reported in previous studies [49,50]. Ampelopsin (**13**) scavenged hydroxyl radicals with EC_50_ value 29.4 ± 4.1 μM in the 1, 10-phenanthroline-Fe(II)/H_2_O_2_ system. Our results showed that the IC_50_ value of compound **13** was about 8 fold of that of BHT.

From these results, it was also possible to make a number of correlations regarding the relationships between the structures of isolated compounds and their hydroxyl radical-scavenging activities. Methyl ester (**2**) appeared to enhance the bioactivity of gallic acid (**1**). Glycosylation of gallic acid enhanced the hydroxyl radical-scavenging activity as with gallotannins **3**~**5**, and bioactivity increased in accordance with the increase of galloyl groups. It was found that the antioxidant activities of gallotannins and phenols decreased in the following sequence: **5** > **3** > **4** > **2** > **1**. It appears that as far as the antioxidant activity is concerned, a galloyl group is essential, while ester and glycoside gallates have greater bioactivity. Comparing the hydroxyl radical-scavenging activities of glycoside gallates (**3** and **4**) with phenol glycoside gallates **6**~**8** revealed that glycoside gallates with 1-phenoxy had higher levels of activity, and the bioactivity decreased in the following sequence: **6**~**8** > **3** > **4**. This suggests that the π-donor of the phenoxy substituent made a greater contribution to the antioxidant activity than the hydroxyl or methoxy in position 1 of the glycoside gallate. The antioxidant activity of phenol glycoside gallates substituted for the benzene ring of the 1-glycosyl group was affected through substitution of methoxy, carboxyl, hydroxyl, or 2′,3′-dihydroxypropyl, and the bioactivity decreased in the following sequence: **7** > **8** > **6** (*i.e.*, 2-methoxy-4(2′,3′-dihydroxypropyl) > 3-methoxy-4-hydroxyl > 3-carboxy-4-hydroxyl). It was found that the antioxidant activities of flavan-3-ols decreased in the following sequence: **11** > **9** > **10** (*i.e.*, 3-*O*-gallate, 5′-OH > 3-OH, 5′-OH > 3-OH, 5′-H). This suggests that the pyrogallol moiety and galloyl group is essential in antioxidant activity. Comparing the hydroxyl radical-scavenging activities of flavan-3-ols (**9** and **10)**, flavonol **12**, and dihydroflavonol **13** revealed that a C2–C3 double bond, 4-carbonyl and a coplanar of phenyl and flavonol had higher levels of activity, and the bioactivity decreased in the following sequence: **12** > **9** > **13** > **10**. It was found that the antioxidant activity of isolated compounds decreased in the following sequence: **12** > **7** > **11** > **8** > **6** > **5** > **3** > **9** > **4** > **13** > **2** > **1** > **10**. This suggests that the *O*-dihydroxy, C2–C3 double bond and 4-carbonyl, and galloyl group increased the hydroxyl radical-scavenging activities.

#### 2.2.3. Peroxynitrite Radical Detection

Peroxynitrite (PON), a potent oxidizing and nitrating species, leads to tissue damage in a number of pathological conditions in humans and experimental animals [51]. Herein, isolated compounds from *C. pulcherrima* pods were examined for their ability to protect against PON-dependent oxidation. Thus, the PON radical-scavenging activities of these isolated compounds were investigated by examining the oxidation of DHR 123. All test compounds had exhibited unappreciable scavenging properties against PON radicals, because none of the polyphenols showed better antioxidative activity than the positive control (BHT) (Table 2). The IC_50_ values for compounds **2** and **10** were 23.42 and 33.10 μM, respectively. The (±) gallocatechin (**9**, 1.63 μM) and (±) catechin (**10**, 1.72 μM) have been reported to provide almost fifty percent inhibition of peroxynitrite and were more effective than penicillamine, a peroxynitrite effective scavenger [52]. In this study, gallic acid (**1**) and (+) gallocatechin (**9**) showed weaker peroxynitrite-scavenging activity than the one obtained (IC_50_ = 24.6 μM) for inhibiting peroxynitrite-mediated tyrosine nitration [53]. (+)-Catechin (**10**) showed bioactivity similar to that reported (IC_50_ = 36.1 μM) in Ketsawatsakul’s study [53].

From these results, it was also possible to make a number of correlations regarding the relationship between the structures of isolated compounds and their PON radical-scavenging activities. Methy ester (**2**) appeared to enhance the bioactivity of gallic acid (**1**). Glycosylation of gallic acid decreased the PON radical-scavenging activity as with gallotannins **3**, **4**, and **5**. It was found that the antioxidant activities of gallotannins and phenols decreased in the following sequence: **2** > **1** > **5** > **4** > **3**. It appeared that as far as the antioxidant activity was concerned, a galloyl group was essential, while methyl gallate had greater bioactivity. Comparing the hydroxyl radical-scavenging activities of glycoside gallates (**3** and **4**) with phenol glycoside gallates **6**~**8** revealed that glycoside gallates with 1-phenoxy had a higher level of activity, and the bioactivity decreased in the following sequence: **6**~**8** > **4** > **3**. This suggests that the π-donor of the phenoxy substituent made a greater contribution to the antioxidant activity than the hydroxyl or methoxy at position 1 of the glycoside gallate. The antioxidant activities of phenol glycoside gallates substituted for the benzene ring of the 1-glycosyl group was affected through substitution of methoxy, carboxyl, hydroxyl, or 2′,3′-dihydroxypropyl, and the bioactivity decreased in the following sequence: **7** > **8** > **6** (*i.e.*, 2-methoxy-4(2′,3′-dihydroxypropyl) > 3-methoxy-4-hydroxyl > 3-carboxy-4-hydroxyl). It was found that the antioxidant activities of flavan-3-ols decreased in the following sequence: **10** > **11** > **9** (*i.e.*, 3-OH, 5′-H > 3-*O*-gallate, 5′-OH > 3-OH, 5′-OH). This suggests that a galloyl group and *O*-dihydroxy (3′,4′-diOH, *i.e.*, catechol) is essential, and 5′-OH is not an important group in antioxidant activity. Comparing the hydroxyl radical-scavenging activities of flavan-3-ol (**9** and **10**), flavonol **12**, and dihydroflavonol **13** revealed that the 4-carbonyl group had a lower level of activity, and the bioactivity decreased in the following sequence: **10** > **12** > **13** > **9**. It was found that the antioxidant activity of isolated compounds decreased in the following sequence: **2** > **10** > **7** > **11** > **8** > **12** > **13** > **6** > **1** > **5** > **4** > **3** > **9**.

## 3. Experimental Section

### 3.1. Chemicals and Reagents

1,1-Diphenyl-2-picrylhydrazyl (DPPH), 2-thiobarbituric acid (TBA), ascorbic acid, butylated hydroxytoluene (BHT), FeCl_3_, 2-deoxy-d-ribose, EDTA, H_2_O_2_, sodium chloride, dihydrorhodamine (DHR) 123, and trichloroacetic acid (TCA) were purchased from Sigma-Aldrich (St. Louis, MO). Dimethylformamide (DMF), methanol, CH_2_Cl_2_, *n*-BuOH, *n*-hexane, ethyl acetate, and acetone were purchased from Merck (Darmstadt, Germany). Peroxynitrite (PON) was synthesized by the ozonolysis of alkaline sodium azide solution as described by Pryor *et al*. [54] (1995). The final PON concentration was determined spectrophotometrically at 302 nm (*ɛ* = 1670 M^−1^·cm^−1^).

### 3.2. General Chemical Experiment

The melting point was recorded on a Büchi B-545 melting-point apparatus and was uncorrected. The optical rotation was measured on a Jasco DIP-1020 digital polarimeter. ^1^H and ^13^C nuclear magnetic resonance (NMR) spectra were obtained on a Bruker AM-500 (500 MHz) FT-NMR spectrometer in d-solvent, using the solvent as the internal standard. The EIMS was determined on a Finnigan TSQ-700 mass spectrometer. The ESIMS was determined on a VG Platform electrospray mass spectrometer. The FABMS was determined on a JEOL JMS-700 mass spectrometer. Column chromatography was carried out with Diaion HP20 (100-200 mesh, Mitsubishi Chemical Industries, Japan), Sephadex LH-20 (20~100 μm, Pharmacia Fine Chemicals, China), MCI-gel CHP 20P (75~150 μm, Mitsubishi Chemical Industries, Japan), and Cosmosil C_18_-OPN (75 μm, Nacalai Tesque, USA). TLC was conducted on silica gel plates (60 F-254, Merck), and a 10% sulfuric acid solution was used as the visualizing agent on heating.

### 3.3. Plant Material

Pods of *C. pulcherrima* were collected from the southern part of Taiwan. Their authenticity was confirmed by Prof. Hsien-Chang Chang (Graduate Institute of Pharmacognosy, Taipei Medical University) using morphological and anatomical techniques. A voucher specimen (TMU-HFL-088) of the plant was deposited at the Herbarium of the Graduate Institute of Pharmacognosy of Taipei Medical University.

### 3.4. Extraction and Isolation

Fresh *C. pulcherrima* pods (8.8 kg) were extracted with 80% aqueous acetone (at a ratio of solvent volume/dry weight of about 2 mL/g) three times, and then concentrated into a residue (1.0 kg) under a vacuum at 45 °C. The residue was subjected to column chromatography over a Diaion HP-20 column (14 × 110 cm) and eluted with a step gradient system (H_2_O–MeOH, 0%~100%) to afford eight fractions of D1~8. Fraction D2 (75 g) was divided into subfractions A1~7 by passage over a Sephadex LH-20 column (10 × 90 cm) and eluted with a step gradient system (H_2_O–MeOH, 10%~100%). Gallic acid [33] (**1**, 0.24 g) and 6-*O*-galloyl-d-glucose [35,36] (**3**, 35 mg) were obtained from fraction A3 (4 g) by MCI-gel CHP 20P column (5 × 70 cm) chromatography, using a step gradient system (H_2_O-MeOH, 10%~100%). Fraction D3 (130 g) was divided into subfractions B1~8 by passage through a Sephadex LH-20 column (10 × 90 cm), eluted with a step gradient system (EtOH-H_2_O, 10%~100%). Fraction B3 (8 g) was further separated by MCI-gel CHP 20P column (5 × 70 cm) chromatography, using a step gradient system (H_2_O–MeOH, 10%~100%) to give subfractions C1~5. Methyl 6-*O*-galloyl-β-d-glucoside [37] (**4**, 100 mg) and gentisic acid 5-*O*-β-d-(6′-*O*-galloyl) glucopyranoside [38] (**6**, 10 mg) were obtained from fraction C2 (2.3 g) by Cosmosil C_18_-OPN column (4 × 55 cm) chromatography, using a step gradient system (H_2_O–MeOH, 10%~100%). Fraction D4 (82 g) was divided into subfractions E1~8 by passage through a MCI-gel CHP 20P column (10 × 90 cm), eluted with a step gradient system (H_2_O–MeOH, 10%~100%). Fraction E3 (20 g) was further separated by Sephadex LH-20 column (5 × 70 cm) chromatography, using a step gradient system (EtOH–H_2_O, 10%~100%) to give subfractions F1~4. Fraction F3 (5.5 g) was divided into subfractions G1~5 by passage through a Sephadex LH-20 column (5 × 70 cm), eluted with a step gradient system (H_2_O–MeOH, 10%~100%). (+)-Gallocatechin [30] (**9**, 0.62 g) and (+)-catechin [41] (**10**, 25 mg) were obtained from fraction G2 (1.2 g) by Cosmosil C_18_-OPN column (3 × 70 cm) chromatography, using a step gradient system (H_2_O–MeOH, 10%~100%). Methyl gallate [34] (**2**, 25 mg) and myricetin 3-rhamnoside [42] (**12**, 0.62 g) were obtained from fraction D5 (65 g) by Sephadex LH-20 column (10 × 90 cm) chromatography, using a step gradient system (EtOH–H_2_O, 10%~100%). Fraction D6 (130 g) was divided into subfractions H1~6 by passage over a Sephadex LH-20 column (10 × 90 cm), eluted with a step gradient system (EtOH–H_2_O, 10%~100%). Fraction H4 (15 g) was further separated by MCI-gel CHP 20P column (5 × 70 cm) chromatography, using a step gradient system (H_2_O–MeOH, 10%–100%) to give subfractions I1~5. Methyl 3,6-di-*O*-galloyl-β-d-glucopyranoside [25,55] (**5**, 0.15 g) and gentisic acid (+)-gallocatechin 3-*O*-gallate [41] (**11**, 13 mg) were obtained from fraction I5 (2.5 g) by Sephadex LH-20 column (4 × 55 cm) chromatography, using a step gradient system (H_2_O–MeOH, 10%-100%). Fraction D7 (210 g) was divided into subfractions J1~8 by passage through a MCI-gel CHP 20P column (10 × 140 cm), eluted with a step gradient system (H_2_O–MeOH, 10%~100%). Three compounds, guaiacylglycerol 4-*O*-α-d-(6′-*O*-galloyl) glucopyranoside (**7**, 28 mg), 3-methoxy-4-hydroxyphenol 1-*O*-α-d-(6′-*O*-galloyl)glucopyranoside [38,39] (**8**, 20 mg), and ampelopsin [43] (**13**, 35 mg) were obtained from fraction J3 (3.8 g) by Sephadex LH-20 column (5 × 70 cm) chromatography, using acetone as the solvent system.

### 3.5. Characterization Data

Guaiacylglycerol 4-*O*-α-d-(6′-*O*-galloyl)glucopyranoside (**7**). Colourless needles; mp 187~190 °C; [α]_D_^20^ −14.1° (c 1.0, MeOH); UV *λ*_max_/nm 277; IR *ν*_max_/cm^−1^ 3368, 1698, 1616, 1227, 1072; for ^1^H and ^13^C NMR spectroscopic data, see Table 1; positive FAB-MS *m/z* 551([M + Na]^+^); HR-FAB-MS *m*/*z* 551.1382 [M + Na]^+^ (calcd. for C_23_H_28_NaO_14_, 551.1377).

### 3.6. DPPH Radical-Scavenging Effect

Various solutions of isolated compounds in methanol (0.5−4.0 μg/mL) were individually added to 0.1 mM of DPPH radical in MeOH according to the method of Hatano *et al*. [56]. The mixture was incubated for 20 min at 37 °C, with the absorbance of the resulting solution measured at 517 nm. The inhibition percentage (*I*%) of the radical-scavenging capacity was calculated using the following equation: *I*% = ((*A*_DPPH_ − *A*_blank_) − (*A*_s-DPPH_ − *A*_s-blank_))/(*A*_DPPH_ − *A*_blank_) × 100; where A_DPPH_ is the absorbance of the DPPH-only solution, *A*_blank_ is the absorbance of methanol instead of the DPPH solution, *A*_s-DPPH_ is the absorbance of the DPPH solution in the presence of sample, and *A*_s-blank_ is the absorbance of methanol in the presence of sample. BHT was used as a positive control. All experiments involving these samples were triplicated. IC_50_ values, which represent the concentration of sample that causes 50% DPPH radical-scavenging activity, were calculated from the plot of inhibition percentage against sample concentration.

### 3.7. Hydroxyl Radical-Scavenging Activity

The hydroxyl radical-scavenging activity was monitored using the 2-deoxyribose method of Halliwell *et al*. [57]. Briefly, the assay mixture contained 2.8 mM 2-deoxyribose, 20 μM ferrous ion solution, 100 μM EDTA, and different sample concentrations in a total volume of 1 mL of 10 mM potassium phosphate buffer (pH 7.4). All components were dissolved in 10 mM phosphate buffer (pH 7.4). The ferrous iron solution and EDTA were premixed before they were added to the assay mixture. The reaction was initiated by the addition of a mixture of 1.42 μM H_2_O_2_ and 100 μM ascorbate. The mixture was incubated at 37 °C for 30 min. At the end of the incubation period, 1 mL of 1% (*w*/*v*) TBA in 50 mM sodium hydroxide and 1 mL of 2.8% (*w*/*v*) TCA were added, the mixture was heated for 30 min in a boiling water bath, cooled, and the absorbance at 532 nm was measured, which corresponds to deoxyribose damage. BHT was used as a positive control. All experiments involving these samples were triplicated. The inhibition percentage (*I*%) of the radical-scavenging capacity was calculated using the following equation: *I*% = ((*A*_hydroxyl_ − *A*_blank_) − (*A*_s-hydroxyl_ − *A*_s-blank_))/(*A*_hydroxyl_ − *A*_blank_) × 100; where A_hydroxyl_ is the absorbance of the hydroxyl-only solution, *A*_blank_ is the absorbance of methanol instead of the hydroxyl solution, *A*_s-hydroxyl_ is the absorbance of the hydroxyl solution in the presence of sample, and *A*_s-blank_ is the absorbance of methanol in the presence of sample. IC_50_ values, which represent the concentration of sample that caused 50% hydroxyl radical-scavenging activity, were calculated from the plot of inhibition percentage against sample concentration.

### 3.8. Peroxynitrite (PON) Radical Detection

#### 3.8.1. Synthesis of PON

PON was synthesized by the ozonolysis of alkaline sodium azide solution for 2 h at 0~4 °C as described by Pryor *et al*. [54]. The PON solution thus prepared was stored at −20 °C and used within 3~4 weeks. The concentration of PON was determined by measuring its absorbance at 302 nm, using an extinction coefficient of 1670 M^−1^ cm^−1^.

#### 3.8.2. Assay of PON-Mediated Oxidation of Dihydrorhodamine (DHR) 123

The PON-induced oxidation of DHR 123 was performed as described by Kooy *et al*. [58]. Briefly, PON (at a final concentration of 10 mM) was added to 50 mM DHR 123 in the absence or presence of different concentrations of test compounds in 0.1 M phosphate buffer containing 0.1 mM diethylenetriaminepenta-acetic acid (DTPA), at pH 7.3, at room temperature. The fluorescence of the oxidized product was measured in a spectrofluorometer (Hitachi F4010) with excitation and emission wavelengths of 500 and 536 nm, respectively. All experiments involving these samples were triplicated.

## 4. Conclusions

Chromatographic separation of 80% aqueous acetone extract of *C. pulcherrima* pods has obtained one new phenol glycoside gallate, and the structure was established as guaiacylglycerol 4-*O*-α-d-(6′-*O-*galloyl) glucopyranoside (**7**). The 12 known polyphenolics were confirmed by referring to the previous literature. All test polyphenolics exhibited moderate to strong radical scavenging properties on DPPH-and hydroxyl radicals, but with weak to moderate scavenging activity toward peroxynitrite radicals in comparison with that of BHT as a positive control *in vitro*. Among them, the most active components belong to the flavonoids. Four compounds, methyl gallate (**2**), methyl 3,6-di-*O*-galloyl-α-d-glucopyranoside (**5**), (+) gallocatechin 3-*O*-gallate (**11**), ampelopsin (**13**), possessed strong DPPH-scavenging activity with IC_50_ values <5.0 μM. For hydroxyl-scavenging activity, the new identified compound, guaiacylglycerol 4-*O*-α-d-(6′-*O*-galloyl)glucopyranoside (**7**) at IC_50_ values <1.50 μM, is capable of exhibiting anti-hydroxyl-radical activity as seen in the presence of (+) gallocatechin 3-*O*-gallate (**11**), and myricetin 3-rhamnoside (**12**). No test compounds showed better activity than BHT. (+)-Gallocatechin 3-*O*-gallate (**11**) is the compound which exhibited excellent activity against both DPPH- and hydroxyl scavenging activity. Flavonoids **9** and **12** are the major compositions, and account for the observed antioxidative effects of the extract of *C. pulcherrima*. To concludee, these results suggest that acetone extracts of the studied *C. pulcherrima* parts possess significant antioxidant. Such radical scavenging activities exhibited by the phytochemical content of the plants make them potential candidates as natural chemoprophylactic agents.

## Figures and Tables

**Table 1:** NMR data for compounds **6**~**8** in acetone-d_6_ + D_2_O.

	6		7		8	
Position	*δ*_C_	*δ*_H_ (mult) (*J* in Hz)	*δ*_C_	*δ*_H_ (mult) (*J* in Hz)	*δ*_C_	*δ*_H_ (mult) (*J* in Hz)
1	119.3		138.0		140.9	
2	156.6		111.9	7.05 d (1.8)	101.7	6.46 d (2.7)
3	117.2	6.64 d (8.8)	149.9		151.7	
4	123.3	7.00 dd (3.1, 8.8)	146.8		154.5	
5	150.0		116.8	7.10 d (8.4)	120.4	7.01 d (8.5)
6	116.2	7.66 d (3)	120.2	6.85 dd (8.4, 1.8)	107.3	6.27 dd (2.7, 8.5)
7			74.4	4.57 d (5.8)		
8			76.9	3.55 ddd (11.2, 5.8, 4.0)		
9			63.9	3.38 dd (11.2, 6.2); 3.47 dd (11.2, 4.0)		
OCH_3_			56.3	3.80 (3H, s)	56.3	3.75 (3H, s)
COOH	174.4					
Glucose						
1′	101.6	4.90 d (7.8)	102.3	4.92 d (7.1)	104.3	4.70 d (7.6)
2′	73.6	3.44 dd (8.0, 9.1)	74.5	3.53 m	74.8	3.51 m
3′	76.1	3.57 t (9.2)	77.7	3.53 m	77.6	3.51 m
4′	70.7	3.32 t (9.5)	71.3	3.53 m	71.2	3.51 m
5′	74.5	3.89 t (9.2)	74.6	3.81 m	75.1	3.70 m
6′	65.4	4.00 dd (8.8, 11.8); 4.63 d (11.6)	64.5	4.32 d (11.8); 4.63 dd (11.8, 1.8)	64.4	4.38 dd (6.1, 11.6); 4.56 dd (2.1, 11.6 )
Galloyl						
1″	119.9		121.6		121.8	
2″, 6″	109.6	7.23 (2H, s)	110.0	7.13 (2H, s)	109.9	7.15 (2H, s)
3″, 5″	145.7		146.1		146.0	
4″	138.9		138.9		138.8	
7″	167.8		166.7		166.7	

**Table 2:** Fifty percent inhibitory concentration (IC_50_) of scavenging activity of phenolic compounds from fresh pods of *Caesalpinia pulcherrima*. (1,1-diphenyl-2-picrylhydrazyl = DPPH, butylated hydroxytoluene = BHT).

Compound	IC_50_ of DPPH radicals	IC_50_ of hydroxyl radicals	IC_50_ of peroxynitrite
BHT	16.57	0.69	0.16
Phenol compounds			
**1**	7.59	8.47	77.23
**2**	4.62	7.45	23.42
Gallotannins			
**3**	7.14	3.90	121.75
**4**	5.38	5.55	101.50
**5**	3.51	2.41	84.38
Phenol glycosides			
**6**	6.53	1.88	76.47
**7**	5.76	0.89	42.84
**8**	6.06	1.74	55.68
Flavan-3-ols			
**9**	12.81	5.26	144.71
**10**	6.38	14.79	33.10
**11**	3.30	1.27	43.69
Flavonol			
**12**	5.14	0.78	57.55
Dihydroflavonol			
**13**	4.94	5.56	73.50
